# Structural, Optical, and Electrical Investigations of Nd_2_O_3_-Doped PVA/PVP Polymeric Composites for Electronic and Optoelectronic Applications

**DOI:** 10.3390/polym15061351

**Published:** 2023-03-08

**Authors:** Samer H. Zyoud, Ali Almoadi, Thekrayat H. AlAbdulaal, Mohammed S. Alqahtani, Farid A. Harraz, Mohammad S. Al-Assiri, Ibrahim S. Yahia, Heba Y. Zahran, Mervat I. Mohammed, Mohamed Sh. Abdel-wahab

**Affiliations:** 1 Department of Mathematics and Sciences, Ajman University, Ajman P.O. Box 346, United Arab Emirates; 2 Nonlinear Dynamics Research Center (NDRC), Ajman University, Ajman P.O. Box 346, United Arab Emirates; 3 Center of Medical and Bio-Allied Health Sciences Research (CMBHSR), Ajman University, Ajman P.O. Box 346, United Arab Emirates; 4 Department of Radiological Sciences, College of Applied Medical Sciences, King Khalid University, Abha 61421, Saudi Arabia; 5 Laboratory of Nano-Smart Materials for Science and Technology (LNSMST), Department of Physics, Faculty of Science, King Khalid University, Abha P.O. Box 9004, Saudi Arabia; 6 BioImaging Unit, Space Research Centre, Department of Physics and Astronomy, University of Leicester, Leicester LE1 7RH, UK; 7 Promising Centre for Sensors and Electronic Devices (PCSED), Advanced Materials and Nano-Research Centre, Najran University, P.O. Box 1988, Najran 11001, Saudi Arabia; 8 Nanomaterials and Nanotechnology Department, Central Metallurgical Research and Development Institute (CMRDI), P.O. Box 87, Helwan, Cairo 11421, Egypt; 9 Department of Physics, Faculty of Science and Arts, Najran University, P.O. Box 1988, Najran 11001, Saudi Arabia; 10 Research Center for Advanced Materials Science (RCAMS), King Khalid University, P.O. Box 9004, Abha 61413, Saudi Arabia; 11 Nanoscience Laboratory for Environmental and Biomedical Applications (NLEBA), Semiconductor Lab., Metallurgical Lab.1., Department of Physics, Faculty of Education, Ain Shams University, Roxy, Cairo 11757, Egypt; 12 Materials Science and Nanotechnology Department, Faculty of Postgraduate Studies for Advanced Sciences, Beni–Suef University, Beni–Suef 62511, Egypt

**Keywords:** PVA/PVP, neodymium oxide-doped PVA/PVP composite films, XRD/FT-IR, nonlinear optical properties, nonlinear *I-V* curve

## Abstract

In this present work, a PVA/PVP-blend polymer was doped with various concentrations of neodymium oxide (PB-Nd^+3^) composite films using the solution casting technique. X-ray diffraction (XRD) analysis was used to investigate the composite structure and proved the semi-crystallinity of the pure PVA/PVP polymeric sample. Furthermore, Fourier transform infrared (FT-IR) analysis, a chemical-structure tool, illustrated a significant interaction of PB-Nd^+3^ elements in the polymeric blends. The transmittance data reached 88% for the host PVA/PVP blend matrix, while the absorption increased with the high dopant quantities of PB-Nd^+3^. The absorption spectrum fitting (ASF) and Tauc’s models optically estimated the direct and indirect energy bandgaps, where the addition of PB-Nd^+3^ concentrations resulted in a drop in the energy bandgap values. A remarkably higher quantity of Urbach energy for the investigated composite films was observed with the increase in the PB-Nd^+3^ contents. Moreover, seven theoretical equations were utilized, in this current research, to indicate the correlation between the refractive index and the energy bandgap. The indirect bandgaps for the proposed composites were evaluated to be in the range of 5.6 eV to 4.82 eV; in addition, the direct energy gaps decreased from 6.09 eV to 5.83 eV as the dopant ratios increased. The nonlinear optical parameters were influenced by adding PB-Nd^+3^, which tended to increase the values. The PB-Nd^+3^ composite films enhanced the optical limiting effects and offered a cut-off laser in the visible region. The real and imaginary parts of the dielectric permittivity of the blend polymer embedded in PB-Nd^+3^ increased in the low-frequency region. The AC conductivity and nonlinear *I-V* characteristics were augmented with the doping level of PB-Nd^+3^ contents in the blended PVA/PVP polymer. The outstanding findings regarding the structural, electrical, optical, and dielectric performance of the proposed materials show that the new PB-Nd^+3^-doped PVA/PVP composite polymeric films are applicable in optoelectronics, cut-off lasers, and electrical devices.

## 1. Introduction

Polymers have become important materials, in recent decades, in various research fields. This is as a result of their attractive characteristics, including flexibility, eco-friendliness, processing simplicity at low temperatures, and inexpensive cost [1]. Moreover, there are fundamental technological applications of the energy-conversion processing of polymers such as solar cells, photovoltaics, and biosensors. On the other hand, the growth in polymeric composite materials obtained from two or more polymer fluctuations suggests a variety of impressive chemical and physical properties [2]. Therefore, long-term durability is vital to the continuous use of composite materials [3]. Additionally, crucial benefits, including corrosion resistance, durability, and faster assembly, are demanded of polymeric composites [4]. These demands have led to the successful doping of rare-earth oxides on composite materials. According to several previously published studies which emphasized the impacts of doping on polymeric composites, there is a need for further research into doping synthesized polymeric composite materials with rare-earth elements [5,6].

The polyvinyl alcohol (PVA) polymer exists within the carbon chain associated with the hydroxyl group. Hence, it exhibits astonishing characteristics such as higher humidity, increased water absorption, and effective synthesized films, which have motivated research into this polymeric blend [5]. Pharmaceutical applications [6], drug coating agents [7], and surgical structures [8] are the kinds of applications that exemplify the importance of PVA. Moreover, PVA photo-cross-linkable gels, hydrogels, and films represent various usages of PVA. For instance, PVA photo-cross-linkable gels were found to form a substrate for enzyme immobilization [9]. In addition, many advantages of PVA gel were detected in the medical field, corresponding to its excellent biosuitability [10]. Furthermore, the growing literature has provided a study on the optical properties of PVA containing several rare-earth sources. For example, Keikhaei et al. investigated the optical properties of PVA with Nd_2_O_3_ using UV-Vis absorption spectroscopy [11]. Meanwhile, polyvinyl pyrrolidone (PVP) is an excellent polymer which was designed to be a coating and additive to several materials [12,13,14]. It is a synthetic polymer created from the radical polymerization of the monomer [15]. Evidence shows that the PVP polymer is, surprisingly, a bulky, non-ionic, non-toxic, temperature-resistant, and biocompatible material [16,17]. High polar amide, polar methylene, and methine groups beyond the ring and backbone of pyrrolidone explain the water-soluble nature of PVP [18]. Although there are extensive reports in the literature on the particular applications extent of PVP, these are restricted to the pharmaceutical [19], cosmetics, and biomedical [16,20] fields.

Due to the successful combination of PVA and PVP polymers, the possibility of establishing a new material has become a reality; their unique properties can be assessed for sophisticated optical and electrical applications [21,22]. Such polymers exhibit structural stability and excellent ionic conductivity [23]. Eco-friendly, easy to fabricate, attractive film-forming, low-cost method, and water-soluble, each of these terms is applicable to a substantial PVA/PVP blend polymer. The corporate PVA/PVP polymeric material matrix leads to a blend polymer. The creative aspiration of developing a new blend polymer begins from the inter-chain hydrogen bonding of a hydroxyl group of PVA and the carbonyl PVP group. Blending polymers affects the crystallinity of PVA doped on PVP [24,25,26]. Future publications report the dramatic existence of lanthanide in PVA/PVP. Junais et al. declared the potential conduction and dielectric relaxation of a PVA/PVP hydrogel synthesized with cerium oxide [27]. Rare-earth oxides have attracted the most interest, exhibiting superb characteristics for various potential applications, including solid UV adsorption, oxygen storage capacity, excellent catalysis luminescence, ceramic industry, and photoluminescence [28,29,30]. Furthermore, neodymium oxide (Nd_2_O_3_) is considered the most substantial rare-earth oxide in the lanthanide series. A treasured feature of the Nd_2_O_3_ rare-earth element has contributed to the development of much more advanced applications such as magnetic devices [31], luminescents [32], photonics [33], thin films [34], and protective coatings [35]. The innovative thermo-luminescent properties of Nd_2_O_3_ exhibit a specific tipping point on gamma-ray dosimetry [36]. Moreover, studies of Nd_2_O_3_ suggest it as a compelling study for rare-earth microwave applications [37] and Nd_2_O_3_ is included in dopings with glass for laser materials [38]. Thin films of Nd_2_O_3_ have added specific values of interest to anti-reflection coatings, gas insulators, and protective coatings [29].

The solution casting process represents a cheap and standard method for producing Nd_2_O_3_-doped PVA/PVP composite films. XRD analysis is used to establish their morphological nature. At the same time, FT-IR characterization is used to examine chemical structures, to study the strong and consistent association between the PVA/PVP blend polymer and Nd_2_O_3_ rare-earth metal oxide. The optical properties of PVA/PVP doping with Nd_2_O_3_ salts are evaluated using UV-Vis-NIR spectroscopy. A new theoretical model verifies the employment of the energy bandgap to estimate the refractive index. Dielectric and electric properties are developed and implemented from the composites’ widely studied dependence on angular frequency and wt%. The exceptional obtained properties of the proposed PB-Nd^+3^-doped PVA/PVP polymeric composites suggest them as excellent candidates for appropriate applications in optoelectronics, cut-off lasers, and electronics.

## 2. Experimental Work

### 2.1. Preparation of PVA/PVP–Nd_2_O_3_ Polymeric Composite Films

**Figure 1:**
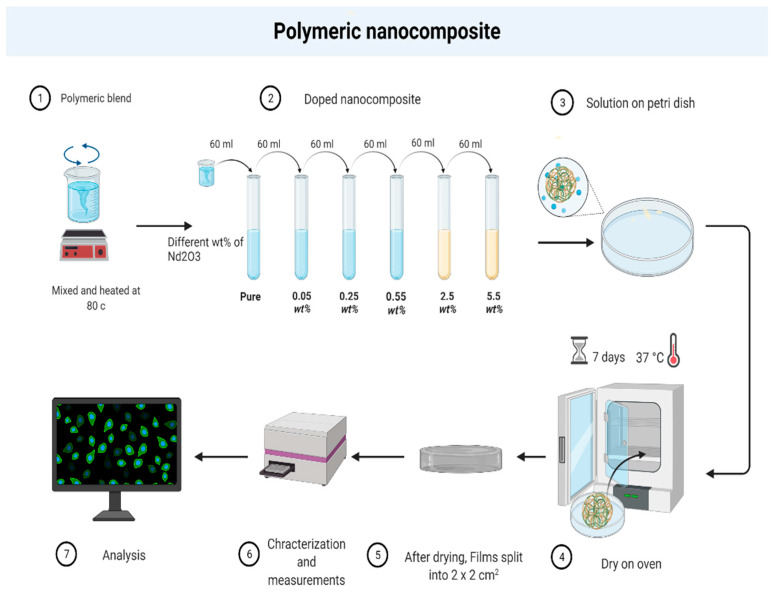
Schematic diagram of the preparation of PB-Nd^+3^ polymeric composites.

Polyvinyl alcohol (PVA), (C_2_H_4_O)_n_ (degree of hydrolysis = 99 percent, molecular weight = 27,000 g/mol); polyvinyl pyrrolidone (C_6_H_9_NO)_n_, average molecular weight 58,000 g/mol; and Nd_2_O_3_ rare-earth elements were supplied from King Khalid University, Abha, Saudi Arabia under number KKU.18-19-1. The low-cost casting process was used to prepare polymeric blend composite films (PVA/PVP–x Nd_2_O_3_); these included x = 0, 0.05, 0.25, 0.55, 2.5, and 5.5 wt%. PVA/PVP films. They were named PB-Nd^+3^-0, PB-Nd^+3^-1, PB-Nd^+3^-2, PB-Nd^+3^-3, PB-Nd^+3^-4, and PB-Nd^+3^-5, respectively, with 70 wt% of PVA—30 wt% PVP composition. The proposed polymeric films were produced using the solution cast technique with a magnetic stirrer at 60 °C for 8 h until a homogenous and transparent solution was obtained. The polymeric composite films of PVA/PVP–x Nd_2_O_3_ were created by adding the necessary Nd_2_O_3_ weight fraction (wt%) to the polymer blend solutions. The Nd_2_O_3_ salt was dispersed uniformly by ultrasonically mixing the solutions. To create free-standing films, the homogenous mixtures were placed into flat Petri plates and left to dry at room temperature for one week. Finally, the obtained Nd_2_O_3_: PVA/PVP polymeric composite films were split into 2 × 2 cm^2^ parts for further investigation, as presented in Figure 1.

### 2.2. Characterizations and Devices

In developing analysis, XRD patterns express the structural morphology of PVA/PVP–x Nd_2_O_3_ thin composite films. XRD data were measured in reflection mode at a scan rate of 0.05 degrees/second using a Shimadzu LabX-XRD-6000 model with *CuK_α_* radiation (*λ* = 1.5406 Å). The operation conditions were a 30 kV voltage and a 30 mA current with the *2θ* range (5–70) in the XRD tube operation. Moreover, the knowledge of the influence of Nd^+3^ content on the functional groups of PVA/PVP was investigated through the transmission spectra in the (400–4000 cm^−1^) wavenumber using FT-IR spectrometer (Thermo Nicolet 6700, Thermo Fisher, Waltham, MA, USA).

JASCO V-570 double-beam spectrometer was utilized to assess the linear optical properties (*T(λ)* and *Abs(λ)* in the light wavelength region between 190 and 2500 nm. The reconstruction of optical measurement was performed at room temperature.

Furthermore, two excitation laser powers were used to evaluate the optical limiting (OL) characteristics of the tested designs. The green laser, the first source, had a wavelength of 532 nm, while the He-Ne laser, the second source, had a wavelength of 632.8 nm with a red beam. Samples were placed at a distance of 0.1 m from the convex lens. Given sufficient consideration, the lens and films were placed at the front of the power photodetector, beyond the line of the incoming laser beam. In addition, superior performers, according to a digitally sensitive laser power meter, were utilized to better understand the results of the output ray.

An automated LCR meter (FLUKE-PM6306 model, Test Equipment Solutions Ltd., Aldermaston, UK) was employed to measure the *AC* electrical conductivity and dielectric properties of the proposed PVA/PVP doping with Nd_2_O_3_ composites. The 100 Hz–1 MHz frequency range was employed at room temperature for measurement assessment. The placement of two copper plates between the samples predicted ohmic contact before measurement. The capacitance (*C*), resistance (*R*), and loss tangent (*tanδ*) of the as-prepared Nd_2_O_3_:PVA/PVP thin films at each excitation frequency, and all of the values, were recorded. In addition, the brass electrode was employed as a sample holder for the measurement of the *I–V* characteristic curve of all as-synthesized Nd^+3^-doped PVA/PVP films.

## 3. Results and Discussion

### 3.1. X-ray Diffraction (XRD) Patterns of PVA/PVP Doped on Nd_2_O_3_ Composites

**Figure 2:**
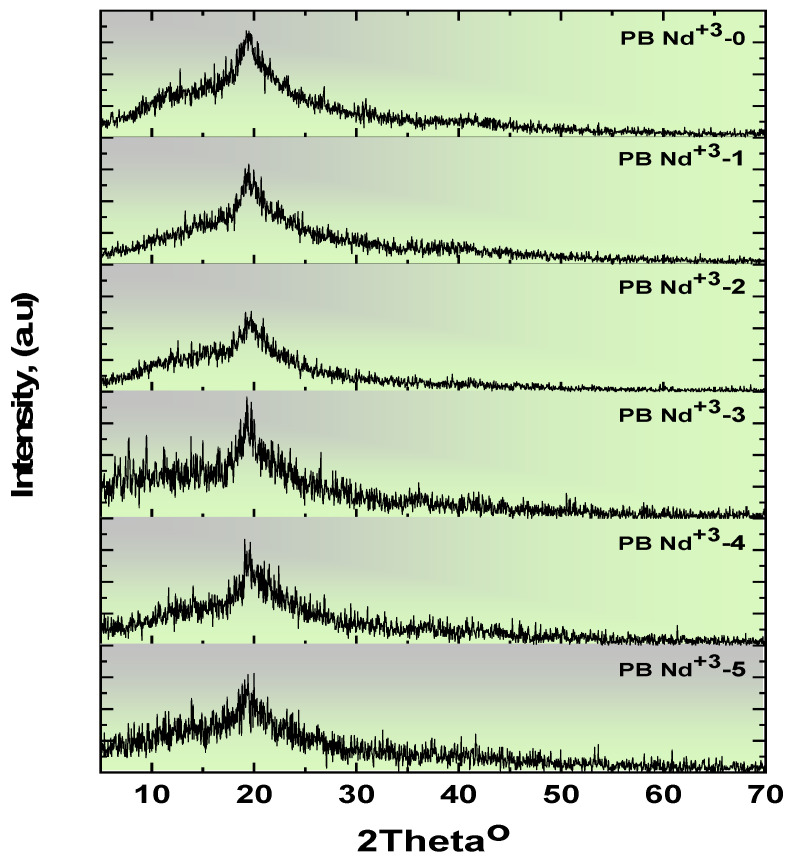
XRD pattern of the polymeric blend doped on composite films with various contents of PB-Nd^+3^.

Accordingly, valuable information was obtained about the structural morphology of the prepared Nd_2_O_3_:PVA/PVP composite polymeric films from the XRD patterns. For instance, Figure 2 displays the XRD pattern of the PVA/PVP polymeric blend doped on Nd_2_O_3_ composite films. In addition, the structural behavior of PB-Nd^+3^ on the polymeric mixture was checked and analyzed. In fact, at the angle of the orthorhombic lattice, regarding the Miller index (101), the diffraction peak was estimated at PVA/PVP at *2θ* = 19.49 [39,40]. Thus, the identified XRD peak is robust and intense.

Furthermore, this XRD peak was in agreement with the semi-crystallinity nature of the PVA/PVP blend polymer, which increased the impact of the incorporation and dispersal of the PVA/PVP intermolecular interaction [40]. However, the dwindling and broadening of the peaks come from the addition of PB-Nd^+3^ to the host PVA/PVP polymeric blend. In addition, the XRD peaks were absent for the high-doping content of PB-Nd^+3^-5. Indeed, its recognizable behavior revealed the high dispersion of PB-Nd^+3^ in the pure PVA/PVP polymeric blend. The most striking findings from these XRD peaks were the composite formation’s location [39,41]. A further result of the investigation was the decrease in the degree of crystallinity, owing to the interaction of hydrogen bonding in the amorphous Nd_2_O_3_-doped PVA/PVP composites. These structural and morphological studies agree well with the XRD results in the literature, from Sadiq et al. [41].

### 3.2. FT-IR Analysis of PVA/PVP Doped on Nd_2_O_3_ Composite Film

**Figure 3:**
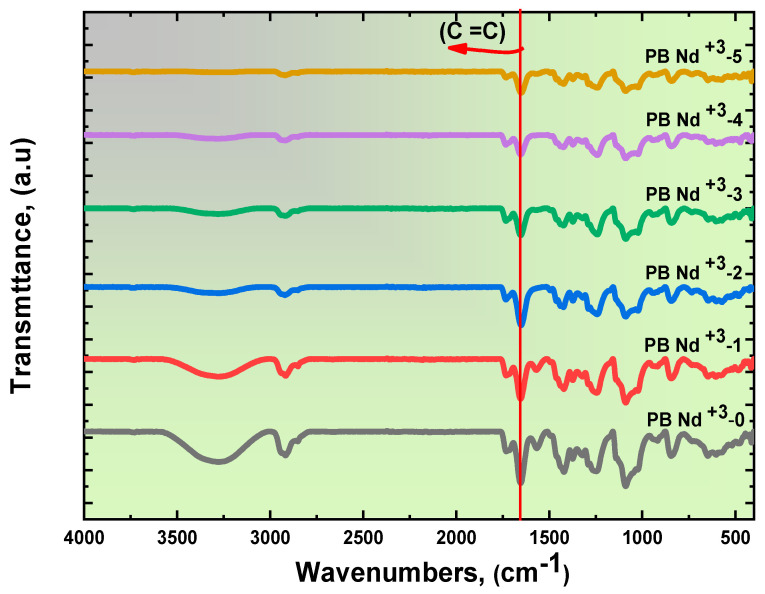
FT-IR transmittance spectra of polymeric blends doped with various contents of PB-Nd^+3^ composite films.

For further investigation, ordinary FT-IR analysis is the most effective method to evaluate the composite films’ structural and chemical characteristics. In addition, FT-IR spectra were employed to identify the infrared absorption and functional group of the Nd^+3^-doped PVA/PVP composite films [42]. The collected FT-IR measurements were in the wavenumber range from 4000 to 400 cm^−1^. Figure 3 shows the FT-IR analysis of the PVA/PVP doped with PB-Nd^+3^ composite films at various concentrations. It was interesting to note, from Figure 2, that the robust band at 3284 cm^−1^ revealed the O-H stretching vibration for the pure PVA/PVP [43,44]. For PB-Nd^+3^-5, the transmittance peak was more pronounced, considering the high wt% content on the PVA/PVP blend polymer. In addition, asymmetric stretching at 2915 cm^−1^ was induced by the CH_2_ group, while the C=C stretching vibration was recorded at 1656 cm^−1^ [44]. In addition, C-H bending was taken as evident in the IR region at 1428 cm^−1^; on the other hand, IR bands at 1090 cm^−1^ were manifested by C=O stretching [44]. The evidence of the precise formation of CH_2_ bending was at the wavenumber 842 cm^−1^ [44].

In contrast, the doped PB-Nd^+3^ established some peaks due to the irregular shift and declined. Nevertheless, the intensity of the peaks can be attributed to variation due to doping. The FT-IR results confirm the link between the PVA/PVP blend polymer and PB-Nd^+3^ composites. There was little agreement between the established FT-IR analysis of the Nd^+3^-doped PVA/PVP polymeric films and the optical data published in the literature by V. Parameswaran et al. for the NH_4_Br in a polyvinyl alcohol/poly (N -vinyl pyrrolidone) blend polymer [44].

### 3.3. The Optical Analysis of Nd_2_O_3_-PVA/PVP Polymeric Composite Film

**Figure 4:**
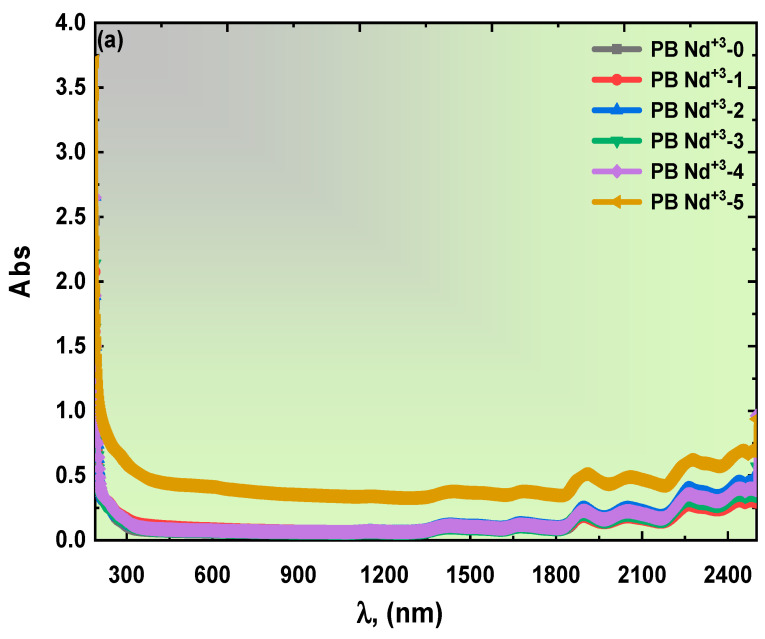

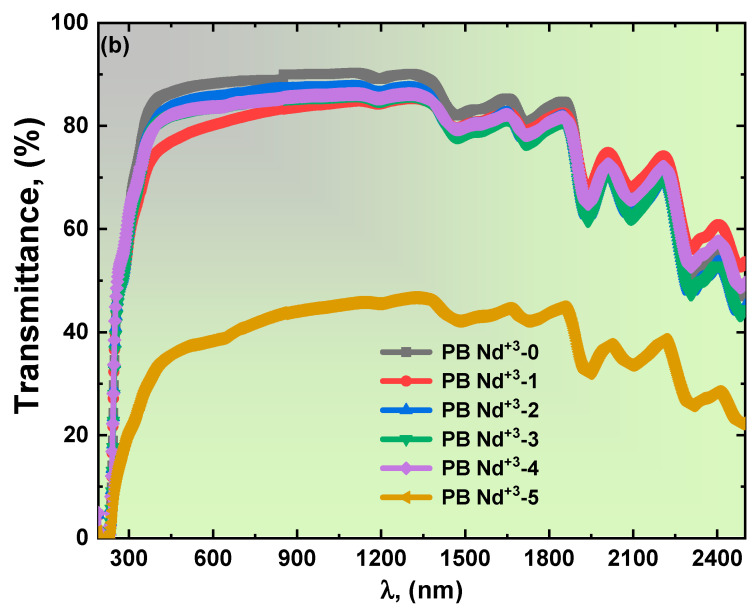
(**a**,**b**): The optical UV-Vis-NIR (**a**) absorbance, and (**b**) transmittance spectra of polymeric blends doped with various contents of PB-Nd^+3^ composite films.

Continuous study has established the UV-Vis- NIR spectrophotometer as the best method of analysis to demonstrate the electronic band, optical behavior, and optical parameters. As illustrated in Figure 4a,b, measurements of the optical spectra for the synthesized Nd^+3^-doped PVA/PVP polymeric composites were reported in the light wavelength range from 190 to 2500 nm. Figure 4a highlights the optical absorption spectra of the PVA/PVP doped with different wt% of the PB-Nd^+3^ composite films. Surprisingly, the absorbance data offered no evidence of the intense peak in the UV- region, while there were multi-oscillation peaks in the IR area. However, the addition of Nd_2_O_3_ at high wt% elevated the absorbance spectrum compared to the pure PVA/PVP blend polymeric sample. In addition, the absorption edge moved to the lower energy state, as it was affected by the high doping content. Perhaps the following absorbance behavior was indicative of the complex charge carrier formation. A similar well-controlled study of prepared Nd_2_O_3_- PVA/PVP composites by A. Hashim [45] agreed with these reported optical results. The blend polymer’s structural, optical, and electronic properties were enhanced through doping with In_2_O_3_ and Cr_2_O_3_ nanoparticles.

Meanwhile, Figure 4b details the optical transmission spectra of the PVA/PVP blend polymer with various doping concentrations of the PB-Nd^+3^ composite films. The maximum transparency inhibited was around 88% for the pure PVA/PVP polymeric matrix, while 36% for (PB-Nd^+3^-5) corresponds to the lowest transmission value. The transmission spectra confirmed the diverse absorption data obtained from the prepared Nd^+3^-doped PVA/PVP composite polymeric films. Here, the precise doping application caused the spectrum to drop in value. This finely tuned action confirmed the correlation of the PB-Nd^+3^ with the composite blend backbone [46]. The obtained linear optical results also persuasively suggested PB-Nd^+3^ as a scattering harbor for the PVA/PVP blend polymer [47].

**Figure 5:**
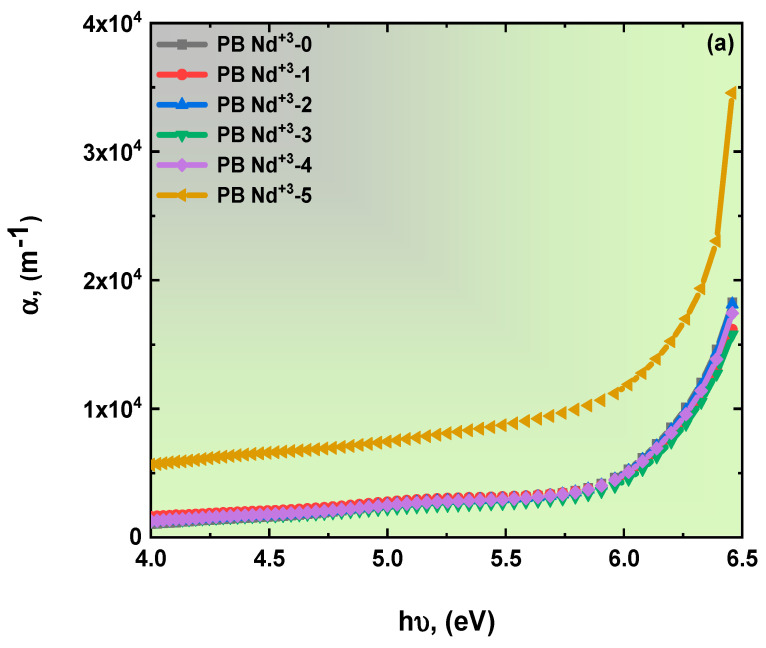

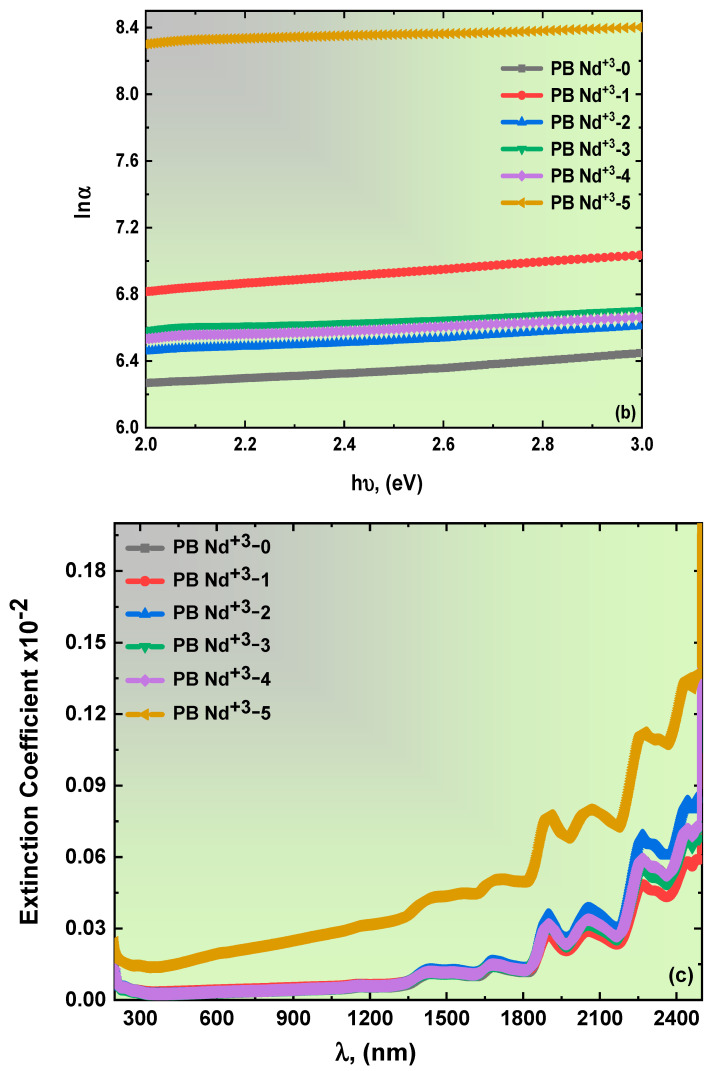
(**a**–**c**): Plots of (**a**) optical absorption coefficient, (**b**) *lnα* versus *hν*, and (**c**) variation in the extinction coefficient related to the wavelength of the blend polymer doped with PB-Nd^+3^ composite films.

The theoretical framework of the absorption coefficient established by Beer’s law suggests a direct relationship between the absorbed radiation and the number of absorbing molecules in the samples [39]. Moreover, the developed parameter offers significant information about unknown energy bandgaps. The following equation represents Beer’s law [48]:

(1) $$\alpha =\frac{2.304\;A\left(\lambda \right)}{t},$$

where *A* presents the absorption, and *t* is the thickness, of the samples. The absorption edge value of the spectrum of PVA/PVP doped with various concentrations of PB-Nd^+3^ composite films is displayed in Figure 5a and Table 1. The quantified and analyzed data of the absorption coefficient indicate the clear impact of the doping of the polymeric blend with a metal oxide (Nd_2_O_3_). The addition of Nd_2_O_3_ caused a shift in the absorption coefficient values of the proposed composites, to the lower energy. Therefore, it also optically affected the energy bandgap values. There is more agreement in Table 1; the absorption coefficient value for the pure PVA/PVP polymer was 5.79 eV, whereas, for PB-Nd^+3^-5, it was 5.47 eV. The above discussion offers significant evidence of the influence of the PB-Nd^+3^ on the blend matrix. One study, so far, provided evidence consistent with these optical results, La^3+^-doped PVA composites by H. Elhosiny Ali et al. [49]. There, the absorption coefficient of the polymeric films decreased with an increase in the doping level. Urbach’s tail width can be used to explore deficiencies in the levels of the unknown bandgap. The following equation indicates the energy of Urbach’s tail [50]:

(2) $$\alpha \left(h\nu \right)=\alpha_{0}\;exp\;\left(\frac{h\nu }{E_{u}}\right),$$

where *E_u_* is Urbach’s tail, (*hν*) is the photon light energy, and *α*_0_ is the energy of the independent constant. The calculated values of *E_u_* were extracted from Figure 5b, as represented in Table 1. It is worth highlighting the contribution of the composite films to raising the value of *E_u_* compared to the pure PVA/PVP blend polymer. The maximum value of *E_u_* contributed to the PB-Nd^+3^-5 value of 12.32 eV. However, the key to the gradually elevated values is increasing the doping level of the PB-Nd^+3^ metal oxide, which created a disturbance which led to dysfunction in the preparation of the composite, and affected the local state of the bandgap [51]. The process attenuated the absorption since the EM waves come through the films, as expressed by the extinction coefficient (*k*), which is expressed through the following equation [52]:

(3) $$k=\frac{\alpha \lambda }{4\pi },$$

**Figure 6:**
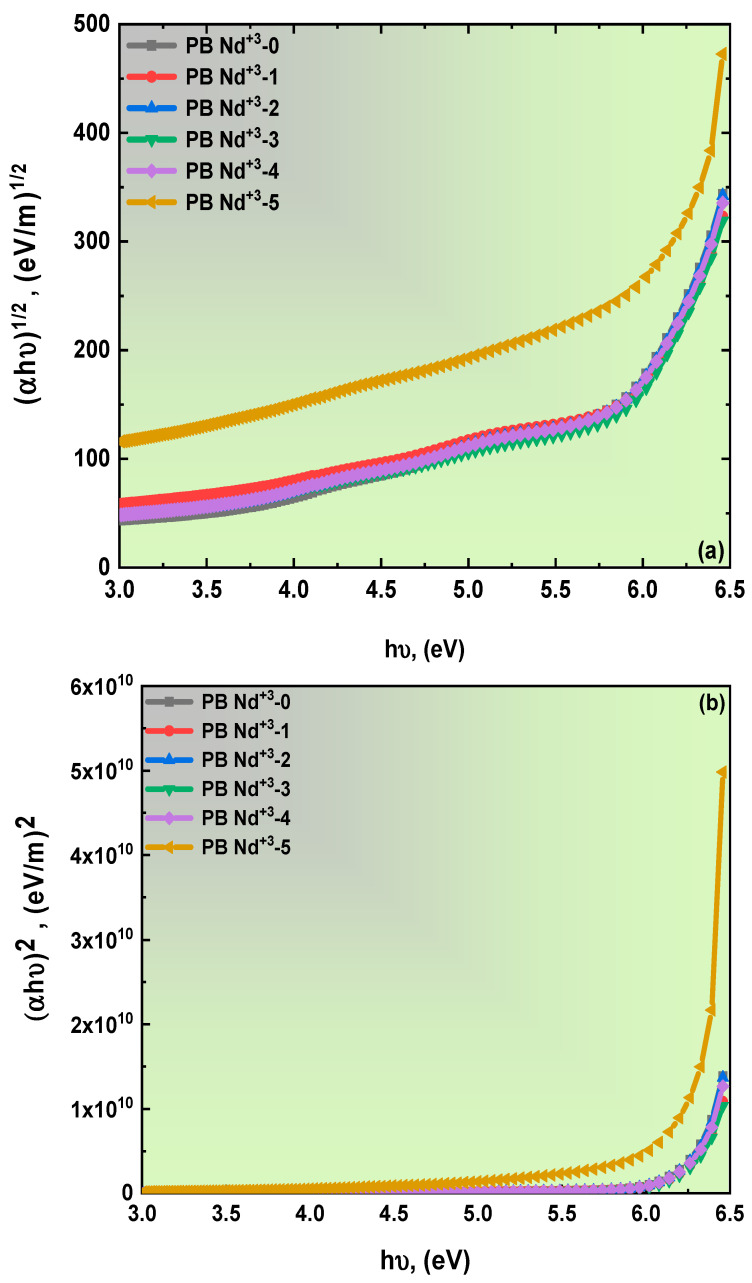
(**a**,**b**): The spectrum of (**a**) *(αhν)*^1/2^ against photons energy *hν*, and (**b**) *(αhν)*^2^ against *hν* for polymer blend doped with PB-Nd^+3^ composite films.

**Figure 7:**
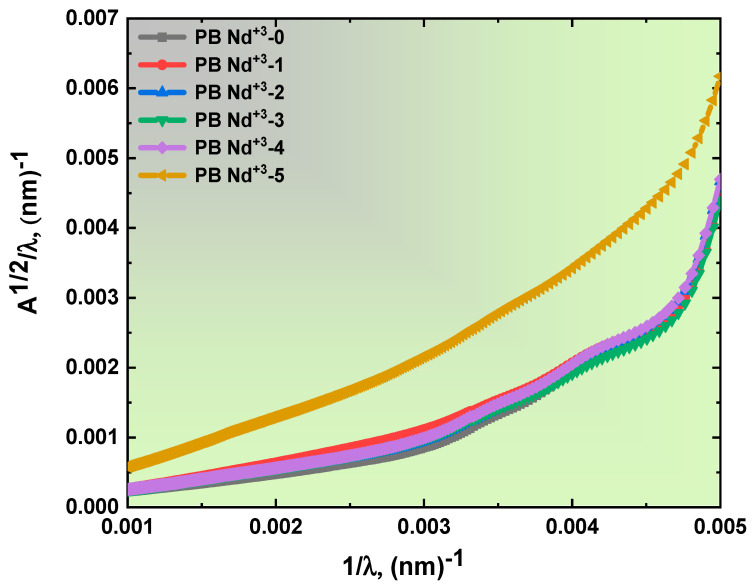
Plot of the variation in *A*^1/2^*/λ* versus 1/*λ* for the PB-Nd^+3^ composite films.

Figure 5c describes a variation in the extinction coefficient as a function of the light wavelength of the blended polymer doped with PB-Nd^+3^ composite film. Figure 5c shows that the *k* increased with the rare-earth doping on the PVA/PVP blend polymer. As a result, the highest value of *k* for PB-Nd^+3^-5 reached 0.18 × 10^−2^. The behavior of the energy optical bandgap defines semiconductors’ ability to insulate materials, corresponding to excellent design and modeling [53]. Hence, the bandgap value was elicited from the transition states from the valence to the conduction band. Tauc’s equation determined the values of the bandgap [54].

(4) $${\left(\alpha h\upsilon \right)}^{\frac{1}{n}\;}=C^{\;}\left(h\upsilon -E_{g}\right)$$

where *C* is constant, and n is a practical value representing a different set; 1/2, 2/3, 2, and 3 indicate the film’s electronic transition. These values expressed the allowed and forbidden direct and indirect transitions. Moreover, a new equation was employed to estimate the energy optical bandgap values. This new calculation relies on absorption spectrum fitting (ASF) without the film thickness. The ASF analysis was performed according to the following relation [55]:

(5) $$A\left(\lambda \right)=D\lambda \left(\frac{1}{\lambda }-\frac{1}{\lambda_{g}}\right)$$

where $D=B{\left(hc\right)}^{m-1}\frac{d}{2.303}$. The leverage of *λg* affirms the specific value of this study, $\;E_{gap}^{ASF}=1240/\lambda_{g}$. The direct and indirect energy bandgaps of the blend polymer doped with PB-Nd^+3^ are depicted in Figure 6a,b, and the variation in the *A*^1/2^*/λ* versus 1/*λ* is present in Figure 7. All the obtained values of the energy bandgap for Tauc’s law and the ASF for the Nd_2_O_3_: PVA/PVP composites are included in Table 1. The recorded direct- and indirect-energy-bandgap values indicate a slight reduction with high doping content. For instance, in the case of the pure PVA/PVP blend polymer, the recorded energy bandgap values were 5.6 for *E_ind_* and 6.09 eV for *E_d_*. By contrast, the value was estimated to be 4.82 eV for *E_ind_* and equaled 5.83 eV for *E_d_* of the PB-Nd^+3^-5 film. In addition, the ASF model’s bandgap exhibited the same behavior as predicted by Tauc’s equation. These bandgap results indicate the creation of defects in the PVA/PVP polymeric matrix [56]. In addition, the decreased values were associated with the formation of charge transfer due to the trap levels identified during the transition between LUMO and HUMO on the blend matrix, resulting in a reduction in the energy required for electron transition [57]. The results suggested the high dispersion of the blend polymer matrix doped with PB-Nd^+3^ composite films. Previous reports on the energy optical bandgap provided confirmation of the suitability of the prepared Nd^+3^–PVA/PVP polymeric composite films [57].

#### 3.3.1. Extraction of the Refractive Index from the Energy Bandgap

Generally, two optical parameters are indicative of a semiconductor’s electronic and optical status, the refractive index and energy bandgap. For instance, semiconductors’ refractive index (*n*) relies on the energy bandgap to deliver outstanding performances in optoelectronic devices, such as a light-emitting diode [58]. However, an indirect relationship was established between these two vital parameters. The high-frequency, static dielectric constant and nonlinear optical properties are directly related to the refractive index. Various empirical equations claim to establish the relationship between the refractive index and the optical energy bandgap. Moss’s equation was the first attempt to express this relation [59]. Moss’s theory divided the solid material’s energy level by the factor of the square root of the dielectric constant, 1/*E_eff_*^2^. The following form depicts the Moss equation [59]:

(6) $$n_{M}^{4}=K/E_{g},\;\;where\;K=95\;eV,$$

Additionally, Ravindra et al. develop the Moss equation by including a new value, *K*. The Ravindra equation calculates the refraction loss by upgrading solar cells’ efficiency [60]. The formula of the Ravindra equation is written as [60]:

(7) $$n_{R}^{4}=\frac{K}{E_{g}},\;\;where\;K\;is\;equal\;to\;108\;eV,$$

After that, based on vibration theory (Penn’s oscillator theory), Hervé and Vandamme proposed the following estimation [61]:

(8) $$n_{H,V}=\sqrt{1+(\frac{13.6}{E_{g}+3.4}}{)}^{2},$$

Meanwhile, Reddy et al. extracted the refractive index by correcting some weaknesses of the Moss equation, as in the following relation [62]:

(9) $$n_{R}=\ln\left(\frac{36.3}{E_{g}}\right),$$

Anani et al. anticipated the given relation of the refractive index [63]:

(10) $$n_{A}=3.4-0.2E_{g},$$

The correlation between the bandgap and refractive index was used to suggest a new empirical equation, by Kumar and Singh. They defined the refractive index as the power law on a bandgap [64]:

(11) $$n_{K}=k\;E_{g\left(dir,ind\right)}^{A},$$

where *A* = −0.32234 and *K* = 3.3668. Recently, the Hosam–Ibrahim–Heba relation employed the Reddy equation to confirm an empirical equation to study the association between the refractive index and bandgap [65]. The study was validated with more than 96 materials, and can be seen in the next relation [65]:

(12) $$n=\sqrt{\frac{A}{E_{g}^{0.5}}}-B,$$

**Figure 8:**
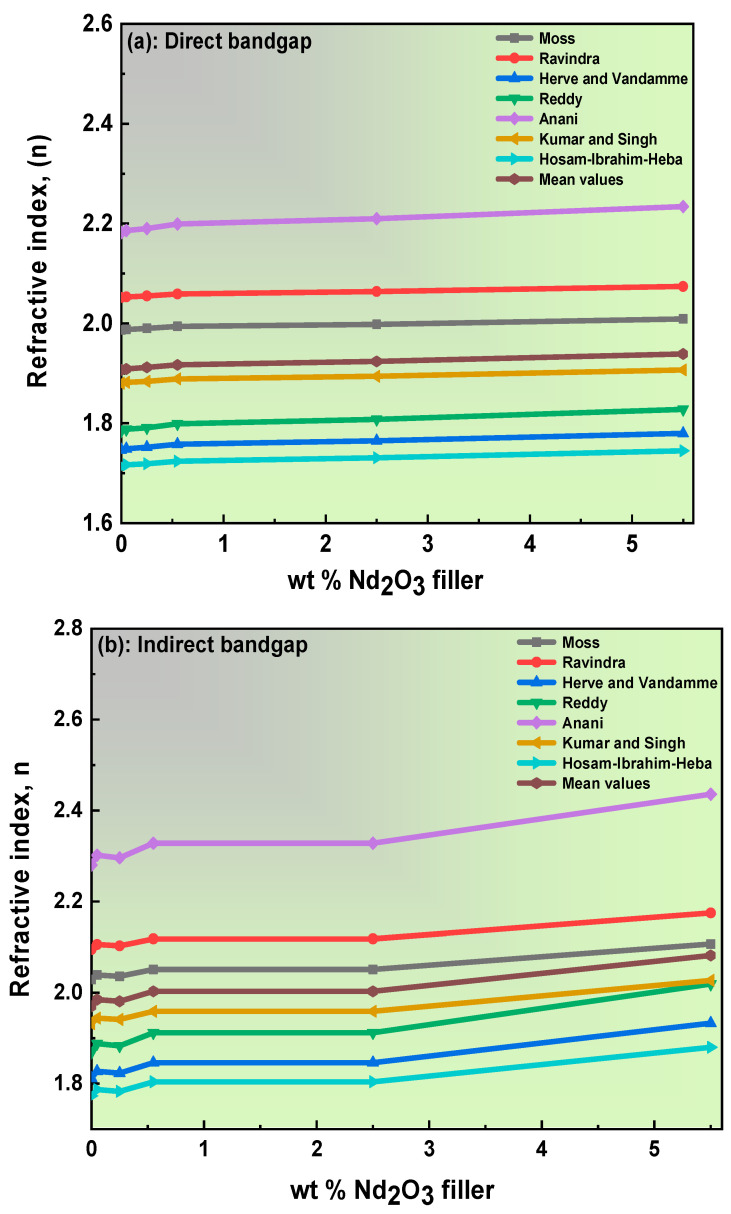
(**a**,**b**): The calculated values of the refractive index (*n*) as a function of (**a**) direct and (**b**) indirect bandgaps for polymer blends doped with PB-Nd^+3^ composite films.

Herein, *A* = 3.442, whereas *B* = √3.44. The deviation values of the refractive index from the experimental equation were calculated and arranged in Table 2. In addition, for more accurate values, the mean refractive index ($n_{AV}$) is detailed in Table 2. In addition, Figure 8a,b shows the deviation in the calculated refractive index as a function of the bandgap of the blend polymer doped with PB-Nd^+3^ composite films. The obtained values become increasingly different from either empirical equation, which is vital to optical applications. As a consequence of adding the PB-Nd^+3^ to the blend PVA/PVP polymer, the refractive index values slightly increase. The great behavior of the values contributes to expanding the reflection, which can help improve free-carrier generation [66]. Furthermore, the capability of creating an anti-reflection surface is among the most influential results from the elevated refractive index values achieved by doping with PB-Nd^+3^ [67]. The average mean value of the refractive index for the pure PVA/PVP polymeric matrix was 1.970; meanwhile, it was 2.082 for the PB-Nd^+3^-5 composite film. These two refraction values established the PB-Nd^+3^ in the blended polymer as a guide for a denser material, and the reduction in the bandgap affected the refractive index value. These complete results are the new yardstick for optoelectronic applications [67].

Ideally, based on the increased refractive index values from the seven equations, the PB-Nd^+3^ filler can be proposed for electronic devices. The high frequency and static dielectric constant were explored by the given equations [68]:

(13) $$\varepsilon_{\infty }=n^{2},$$

(14) $$\varepsilon_{0}=-33.26876+78.61805E_{g}-45.70795E_{g}^{2}+8.32449E_{g}^{3},$$

Table 3 includes the values of the high-frequency and static dielectric constant of the blend polymer doped with PB-Nd^+3^ composite films. The high-frequency and static dielectric constant values of the proposed polymeric films increased with increased Nd^+3^ doping content. The success of doping on the PVA/PVP matrix explains the increased importance of the two parameters of the dielectric constant.

#### 3.3.2. Nonlinear Optical Properties of Nd_2_O_3_-Doped PVA/PVP Composite Films

The reality of a nonlinear attitude becomes evident as the light emerging from the solid material reduces. Directly, the material’s polarization significantly depends on the strength of the electric field. Here, the distributed light on the solids leads to the representation of polarization, with the electric field as a nonlinear fraction. Thus, investigating the nonlinear refractive index (*n*_2_), linear susceptibility $\left(\chi^{\left(1\right)}\right)$, and third nonlinear optical susceptibility $\left(\chi^{\left(3\right)}\right)$ of the solid films is very significant. For instance, the direct association of the polarization with the electric field is expressed by the given equation [69]:

(15) $$P=\chi^{\left(1\right)}\;E+P_{NL},$$

where

(16) $$P_{NL}=\chi^{\left(2\right)}E^{2}+\chi^{\left(3\right)}E^{3},$$

Herein, *P_NL_* is the nonlinear polarizability of the material. By extension, the refraction coefficient *n(λ)* could be defined by the following relation [70]:

(17) $$n\left(\lambda \right)=n_{0}\left(\lambda \right)+n_{2}\left(E^{2}\right),$$

The higher collective values of the linear refractive index *n*_0_(*λ*) can be compared to the nonlinear refraction, since the nonlinear optical parameter is considered an encouraging characteristic for optical communication and the transformation of data, etc. Thus, the linear optical susceptibility is established by the following equation [71]:

(18) $$\chi^{\left(1\right)}=\frac{n_{AV}{}^{2}-1}{4\pi },$$

The third-order nonlinear optical susceptibility $\left(\chi^{\left(3\right)}\right)$ can be determined according to the following equation [72]:

(19) $$\chi^{\left(3\right)}=A\;{\left(\chi^{\left(1\right)}\right)}^{4},$$

The nonlinear refraction index is established by the following formula [73]:

(20) $$n_{2}=\frac{12\pi \chi^{\left(3\right)}}{n_{AV}},$$

The distributed values of $\left(\chi^{\left(1\right)}\right)$, $\left(\chi^{\left(3\right)}\right),$ and *n*_2_ are illustrated in Table 3, where the calculation considered the restricted direct and indirect bandgaps. The representatives of the database are recorded in Table 3, and optically confirmed that the values of the nonlinear parameters were exposed to a consequent increment with the addition of PB-Nd^+3^ to the PVA/PVP polymer. The apparent similarities in the performance of the nonlinear refraction index and the third nonlinear susceptibility are due to their sequence relation. For example, concerning the direct bandgap, the optical values of $\chi^{\left(1\right)}$ ranged from 0.209 esu to 0.220 esu, while $\chi^{\left(3\right)}$ varied between 3.30 × 10^−13^ esu and 3.984 × 10^−13^ esu, and *n*_2_ values ranged from 0.65 × 10^−11^ esu to 0.773 × 10^−11^ esu. The obtained values of the nonlinear optical parameters for the synthesized Nd^+3^-doped PVA/PVP polymeric composites appear to coordinate with the reported data by H. Elhosiny Ali et al. [74,75]. The absorption and linear/nonlinear properties of polyvinyl alcohol (PVA) polymeric thin films are optically enhanced through doping with fullerene [74]. Corresponding to the conducting results, significant attention has been paid to the material’s ability to be employed in nonlinear optical devices [75]. The existence of PB-Nd^+3^ is set to improve the nonlinear optical parameters when doped on the host PVA/PVP matrix.

### 3.4. The Optical Limiting Effects of PVA/PVP Doping with Nd_2_O_3_ Composite Films

**Figure 9:**
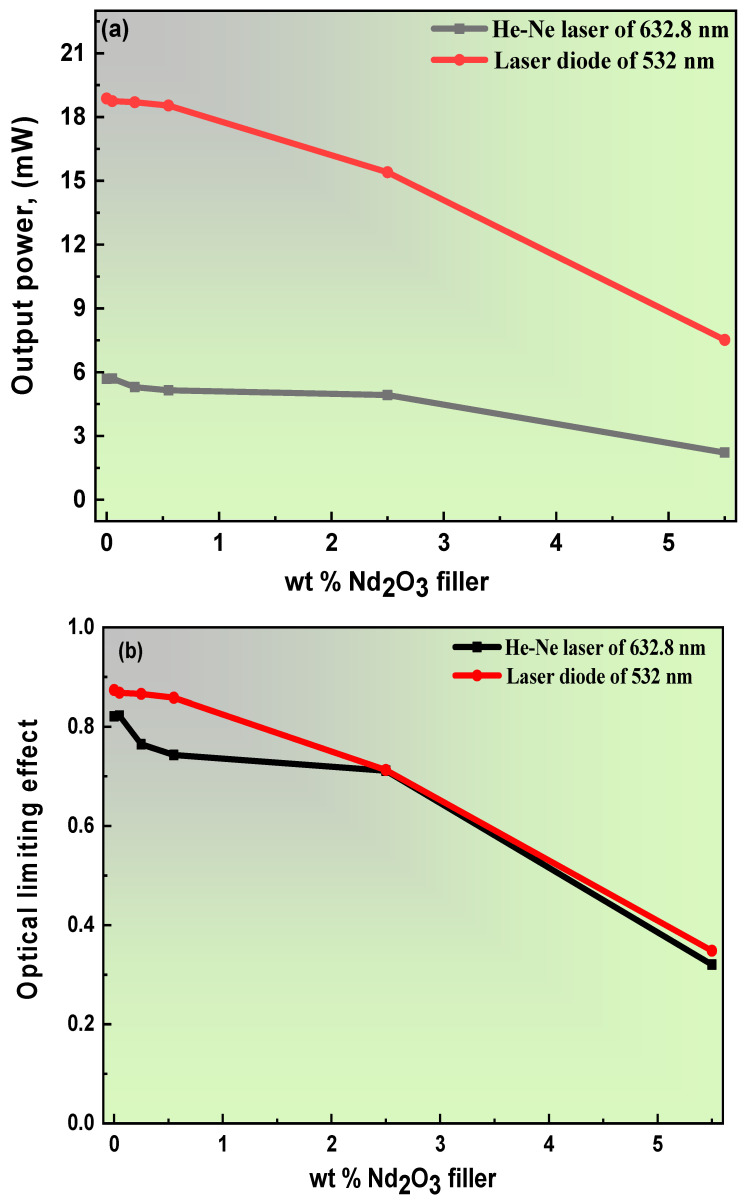
(**a**,**b**): (**a**) output power, (**b**) optical power limiting effects measured with two laser sources, He-Ne at 638.2 nm and diode at 532 nm for Nd^+3^-doped PVA/PVP composites.

The importance of establishing the optical power limiters arises from the valuable benefits of eye protection and equipment of an optically delicate nature. However, more vital information is provided by the optical power limiter, since it is proposed to be primarily responsible for reducing the density from radiation laser power [76]. Herein, two crucial laser powers, a red laser of (632.8 nm He-Ne) and a green laser of 532 nm, were used to identify the optical laser power of the PVA/PVP doped with a Nd_2_O_3_ composite film. Figure 9a,b demonstrates the optical power and limiting of the PVA/PVP doped with PB-Nd^+3^ composite films. Clear evidence shows the contribution of the PB-Nd^+3^ on the blend matrix: it minimizes the output power given a share in the high nonlinear absorption.

Furthermore, as shown, the concentration affects limitation capacity. The response of the optical limiter to a high content to become weaker; meanwhile, a lower content results in high limitation capacity. The reason for this behavior is the high molecule per unit volume of the high doping concentration [77]. The behavior previously confirmed by the laser attenuation, Figure 9b, suggests a CUT-OFF in the visible light. Consequently, the optical limitation efficiency somewhat predicts the transmission value of the film within the same wavelength as the light entering. In particular, the cut-off laser device seeks to address promising applications for the film polymer when the transmission value is exactly or close to zero. Different studies have been able to reproduce these findings [77].

### 3.5. Dielectric Properties of PVA/PVP Doped with Nd_2_O_3_ Composite Films

**Figure 10:**
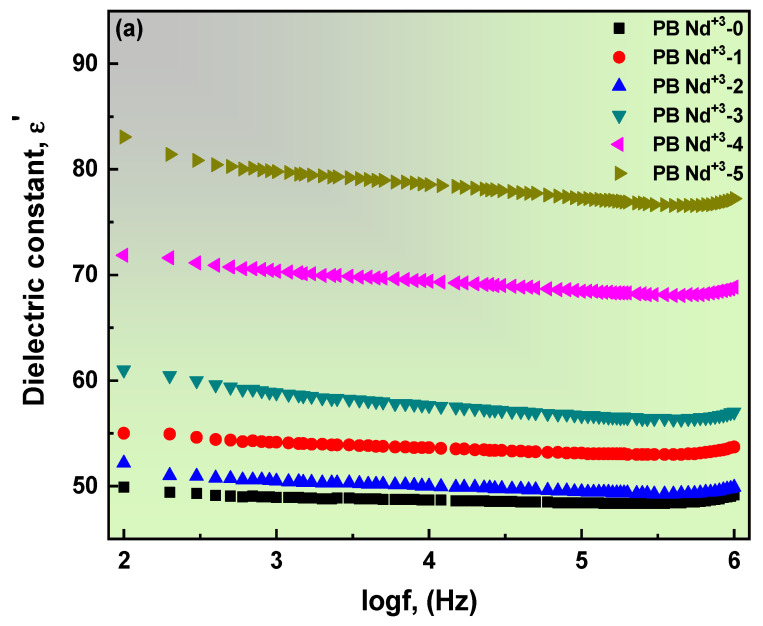

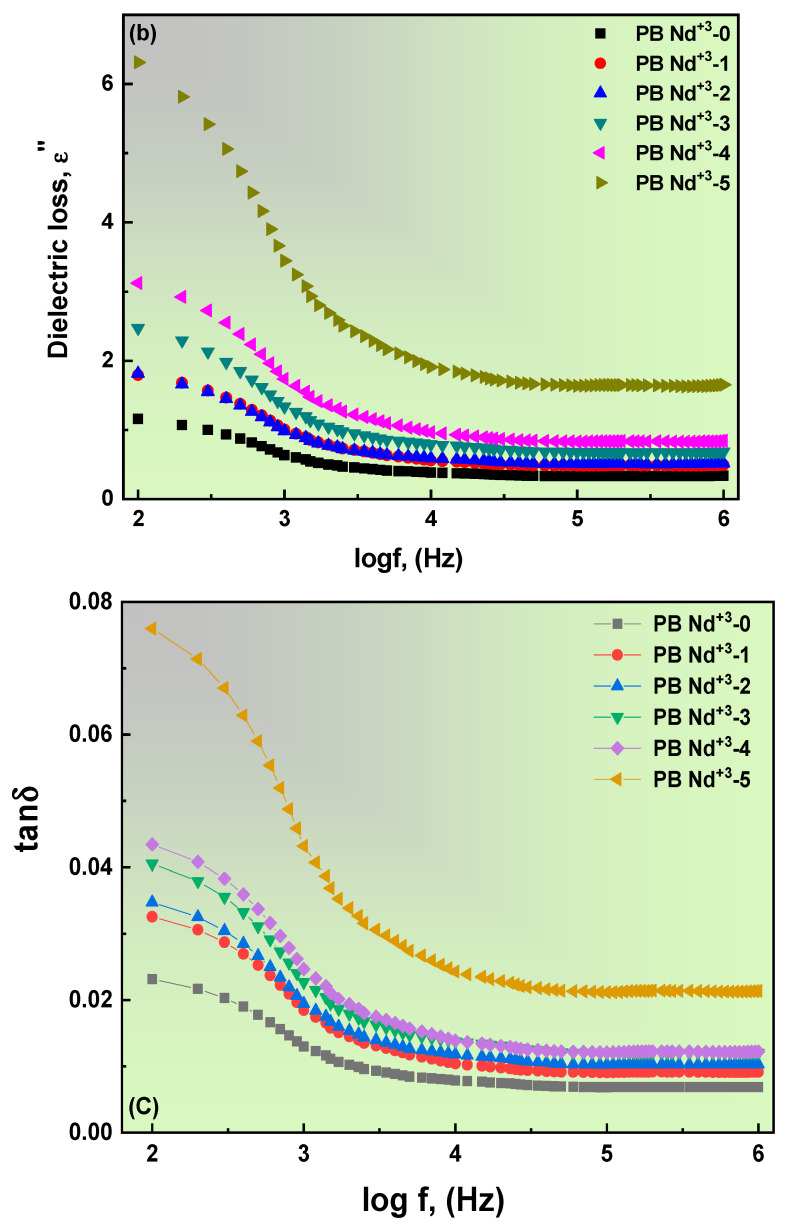
(**a**–**c**): (**a**) Real, (**b**) *tanδ*, and (**c**) imaginary parts of dielectric permittivity against angular frequency for PB-Nd^+3^ composite films.

An insight into the dielectric-behavior study of the composite films establishes the material’s capacitance to store a charge. Furthermore, the investigation of relaxation and conduction establishes the importance of dielectric analysis. Thus, the formulas for calculating the real, imaginary, and tangent represented in these relations are [78,79]:

(21) $$\varepsilon^{\prime }=\frac{C_{p}d}{A\varepsilon_{0}},$$

(22) $$\varepsilon^{\prime \prime }=\frac{d}{2\pi fA\varepsilon_{0}},$$

(23) $$tan\theta =\frac{\varepsilon^{\prime \prime }}{\left[\varepsilon^{\prime }\right]},$$

where *C_p_* is the capacitance; *d* is the thickness; *A* is the area of the electrode plate; and $\varepsilon_{0}$ represents the absolute permittivity of absolute space, which is equal (8.85 × 10^−12^ F/m). Figure 10 depicts the real (a) and imaginary (b) parts of the dielectric permittivity, and (c) the tangent of loss angle (*tanθ*) of the blend polymer doped with the PB-Nd^+3^ composite films. Figure 10a–c demonstrates the significant trend of reduced dielectric constants with frequency increases. The high frequency reveals a diminished space charge polarization within the binding with an electrode. The *ε′* convincingly shows the correlation with PB-Nd^+3^ wt%, which indicates the drop in the ion–ion interface and high conductivity [80]. The elevated *ε′* is commonly associated with increased carrier mobility and concentration. Dipoles and polarization relate to the *ε′* permittivity, establishing an increase in the particle size with the high PB-Nd^+3^ content [67].

Moreover, imperfections and deficiencies may exist, causing the loss of the dielectric properties of the blend polymer. The importance of *ε′* in electronics and insulating materials is established. However, the dependence on frequency is revealed with the permittivity of *ε″*. At a high frequency, the *ε″* permittivity is low; this pattern provides convincing evidence of the minor participation of ionic polarization at this frequency. Meanwhile, the production of the polarization mechanism is discovered at lower frequencies. Including PB-Nd^+3^ increases the *ε″* permittivity values, which rely on increased ionic carriers and conductivity [81]. The existence of rotating polar bonds is related to conductivity. The *tanθ* represents the same behavior as the *ε″* permittivity. The increase in the Nd^+3^ filler on the host PVA/PVP blend polymer produced high values of *tanθ*. The spectrum depicted no lose of peaks corresponding to the conduction, which could obscure the dielectric relaxation. This much broader perspective highlights the relevance of this study.

**Figure 11:**
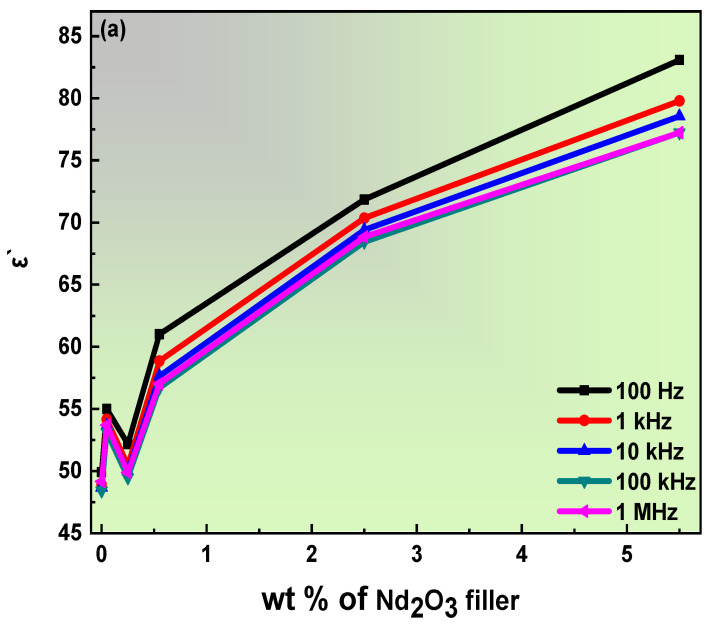

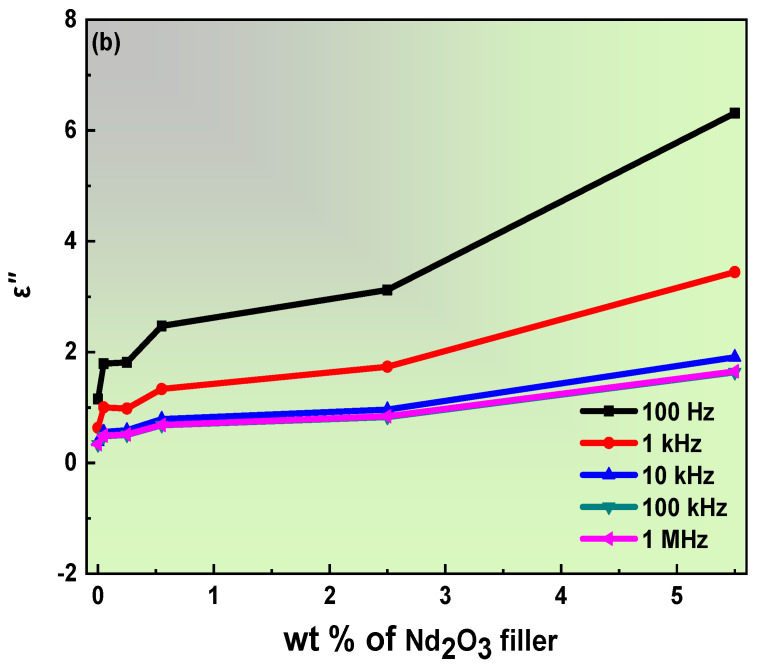
(**a**,**b**): Dependence of (**a**) *ε′* and (**b**) *ε″* values on (wt%) concentration of PB-Nd^+3^ composite films at 30 °C.

Figure 11a,b rules out a correlation between the dielectric permittivity of a given selective frequency and the wt% of the PB-Nd^+3^-doped blend polymer at a temperature of 30 °C. Immediately noted from Figure 11 is the increase in the dielectric permittivity in case of a decrease in the frequency values. The *ε′* values offer a linear connection with impulse; there is a dramatic increase in the values following the addition of PB-Nd^+3^ content to the PVA/PVP composite films. In addition, the estimated value of *ε′* permittivity at 1 MHz is less than 2.5. Therefore, the blend polymer doped with PB-Nd^+3^ films can be approved for participation in the manufacturing of microelectronic and electrical devices such as substrates and insulators, based on this *ε′* low permittivity value [82]. Figure 11b draws attention to the higher *ε″* values compared to *ε′* values. Initially, the lower frequency represents the high value of *ε″*, where the shift in the *ε″* maximum values is suggested following the doping of PB-Nd^+3^ on the blend polymer. Meanwhile, the massive inequality between *ε″* and *ε′* values motivated consideration of a PVA/PVP doped with PB-Nd^+3^ composite films as an effective method for energy storage in micro capacitors [82]. A current study by S. Choudhary demonstrated similar dielectric properties for PVA/PVP blend polymeric composites in flexible nanodielectric devices [82].

### 3.6. Electric Module Study of PVA/PVP Doped with Nd_2_O_3_ Composite Films

Typically, the term electric modules M*(ω) explains the influence of electrode polarization (*EP*) on the dielectric permittivity presented at low-frequency values. The following relation establishes the formula of M* [83]:

(24) $$M^{*}=M^{\prime }+jM^{\prime \prime }=\frac{\varepsilon^{\prime }}{{\varepsilon^{\prime }}^{2}+{\varepsilon^{\prime \prime }}^{2}}+j\frac{\varepsilon^{\prime \prime }}{{\varepsilon^{\prime }}^{2}+{\varepsilon^{\prime \prime }}^{2}},$$

**Figure 12:**
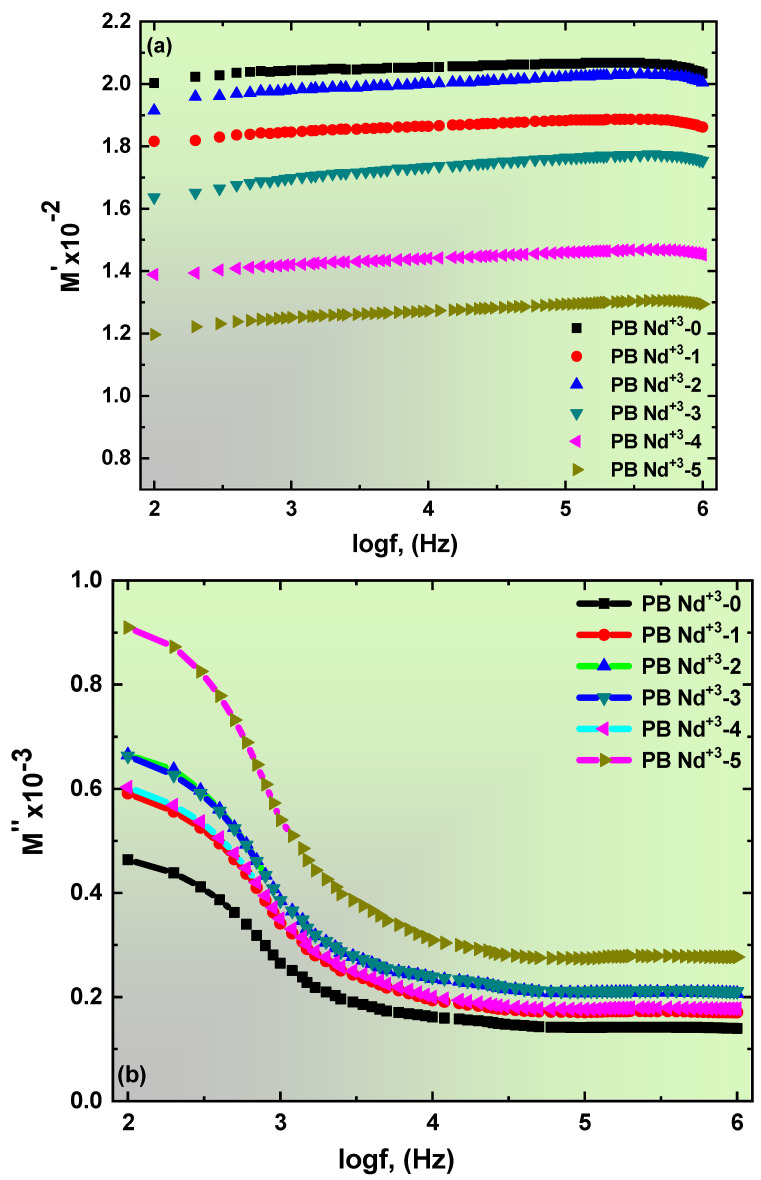
(**a**,**b**): (**a**) Real part of electric modulus (*M′*), (**b**) imaginary part of electric modulus (*M″*) versus frequency for samples at room temperature.

Figure 12a,b highlights the real $M^{\prime }$ and imaginary $M^{\prime \prime }$ parts of the electric modules of the blend polymer doped with PB-Nd^+3^ composite films at room temperature. The constant behavior of the $M^{\prime }$ values for the proposed composites offered at high frequencies is shown in Figure 12a. Therefore, the $M^{\prime }$ pattern of the obtained spectra was nonlinear to that of the $\varepsilon^{\prime }$ permittivity spectra. In addition, there was no peak on the $M^{\prime }$ spectra. These findings contributed to the realization that the lowest value of $\varepsilon^{\prime }$ is achieved a higher frequency, while the maximum value of $M^{\prime }$ is in the high-frequency region of $M_{\infty }=\frac{1}{\varepsilon_{\infty }}$. Furthermore, the addition of the PB-Nd^+3^ concentration to the PVA/PVP blend polymer causes a slight reduction in the $M^{\prime }$ values. Figure 12b shows the $M^{\prime \prime }$ values of the Nd_2_O_3_-doped PVA/PVP composite films. Similarly, identical behavior of the $M^{\prime \prime }$ and $\varepsilon^{\prime }$ spectra was observed for all synthesized polymeric composites. The Nd_2_O_3_: PVA/PVP composite films had no relaxation peaks in lower frequency regions. Therefore, the disappearance of peak relaxation in the low-frequency area was because of the interaction of the filler on the PVA/PVP blend matrix. Furthermore, the $M^{\prime \prime }$ values tended towards the maximum and shifted to the lower frequency region as the doping level of PB-Nd^+3^ increased on the host PVA/PVP polymer. Thus far, these results suggested a significant turn towards a diminished relaxation time and increased ionic conductivity [84]. These electrical results were relevant to the literature, for example, Choudhary [82].

The central focus of the electrical measurements was to confirm the conduction mechanism of Nd^+3^-doped PVA/PVP composite polymeric films, which constitutes a new electrical examination approach. The equations to calculate the *AC* conductivity for the as-prepared doped PVA/PVP blend polymeric films are provided below [85]:

(25) $$\sigma_{Total.AC}\left(\omega \right)=\frac{t}{ZA},$$

(26) $$\sigma_{Total.AC}\left(\omega \right)=\sigma_{DC}\left(\omega \to 0\right)+\sigma_{AC}\left(\omega \right),$$

(27) $$\sigma_{AC}\left(\omega \right)=A\omega^{s},$$

**Figure 13:**
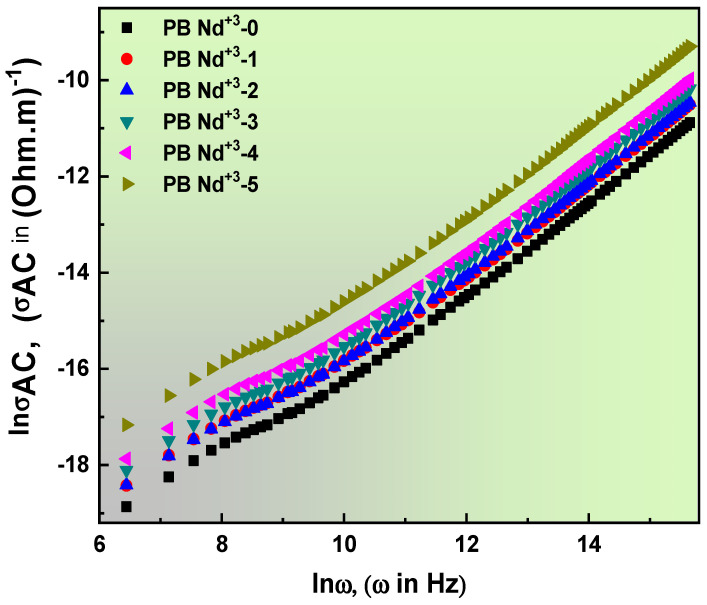
*AC* conductivity vs. angular frequency for different systems with various wt% of PB-Nd^+3^ composite films.

Herein, $\sigma_{Total.AC}$ is represented by the *AC* conductivity, the impedance is *Z*, and *A* is the constant-value variable according to the temperature. Equation (26) is known as the *DC* and *AC* electrical conductivities. In addition, in Equation (26), the angular frequency is presented by ω, and *s* is the frequency exponent parameter. A linear relation defines the *AC* conductivity with applied frequency in Figure 13. The contribution of charge-carrier mobility is a possible result of this finding. With the addition of the PB-Nd^+3^ dopant, the *AC* conductivity pattern increased to its high maximum value. A good summary of the contribution of dopants suggests a higher number of free ions [86]. Moreover, the effectiveness of the applied frequency is established by the extreme values of *AC* conductivity in high-frequency regions. In contrast, the *DC* values extracted from Equation (27) estimated values of $\sigma_{DC}$ induced from the spectra of *AC* conductivity. Table 4 lists the values of $\sigma_{DC}$ according to the extent of PVA/PVP doping with PB-Nd^+3^ composite films.

Nevertheless, the frequency exponent (*s*) can be used to demonstrate the cross-section correlation between material impurities and charge carriers. The *s* values of the prepared Nd^+3^-doped PVA/PVP composites varied from zero to one. From Table 4, it can be seen that s values decreased with an increase in the doping level on the host PVA/PVP matrix. This significant analysis offered the precise coordination of s behavior with many hopping systems. Therefore, this establishes a correlated hopping system (CHF) controlled by the conduction mechanism [87]. The listed values of the *s* exponent ranged from 1.006 to 0.996, confirming the disordered nature of the dielectric medium [88]. However, there is a feasible network route in the Nd^+3^-doped PVA/PVP composite films, due to the increase in the charge carrier on the polymer matrix, as almost the same results were previously reported [89].

### 3.7. I-V Characteristic Plot of PVA/PVP Doping with Nd_2_O_3_ Composite Films

**Figure 14:**
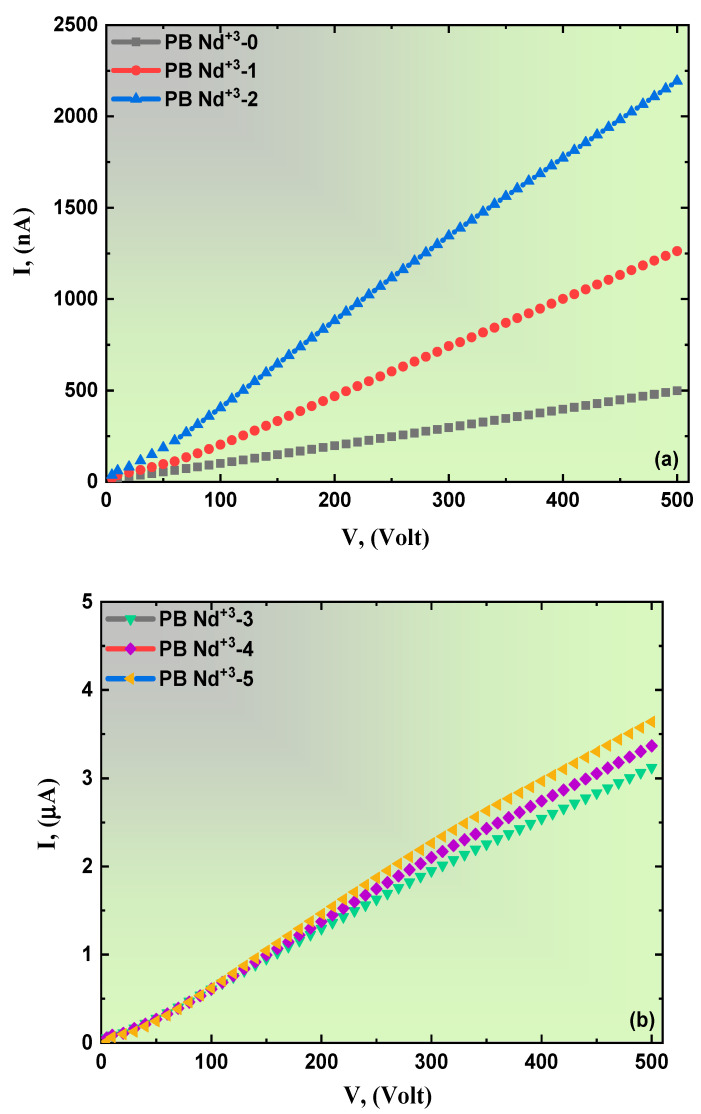

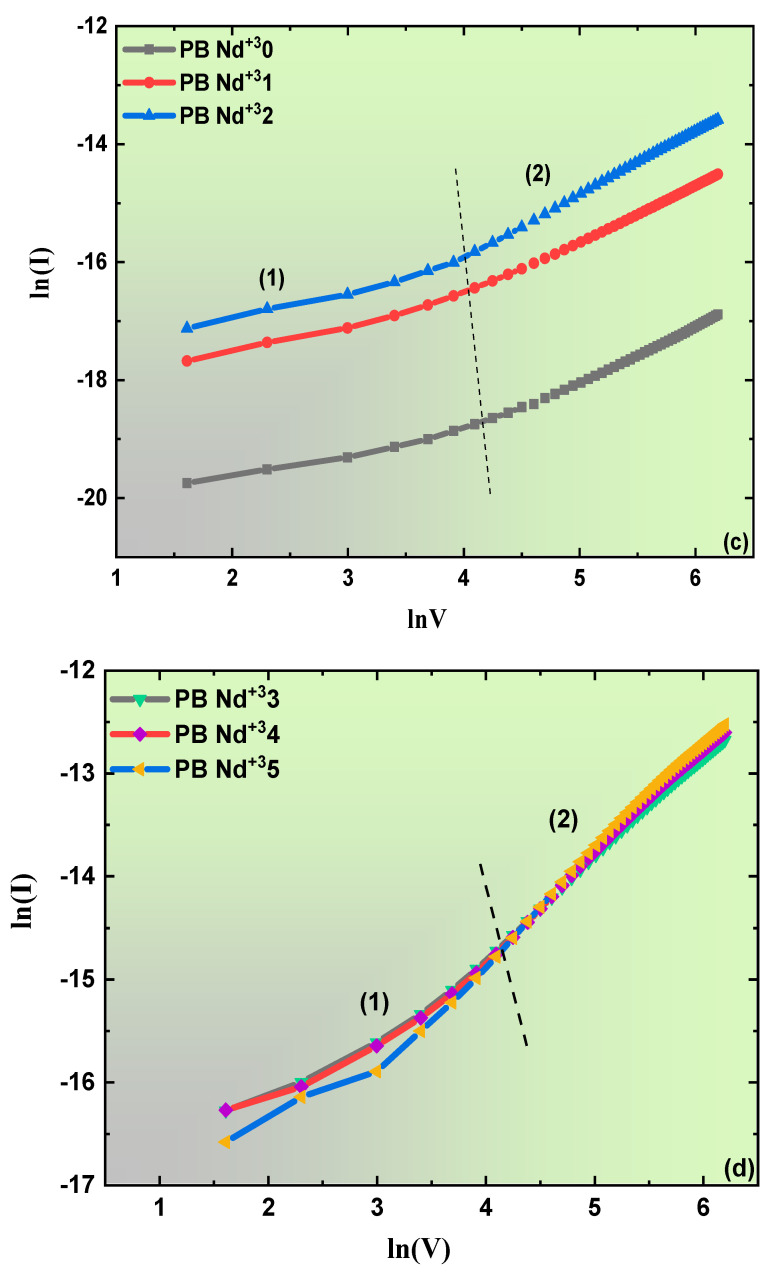
(**a**,**b**): Variation in the current (*I*)-voltage (*V*) plots and (**c**,**d**) the corresponding *ln(I)*-*ln(V)* plots of the PVA/PVP blend polymer doping with PB-Nd^+3^ composite film.

The majority of voltage-dependent resistance can be traced to the development of the massive transient biasing of the elements in the electronic circuit and is designed to protect the components. This is established by a series number of nonlinearly current-voltage properties. Figure 14a,b exhibit I-V nonlinear plots of the PVA/PVP blend polymer films doped with various concentrations of PB-Nd^+3^ rare-earth elements. A high current value was noticed after introducing the significant amounts of PB-Nd^+3^ to the host polymeric matrix. However, the tendency of the pure PVA/PVP film was high ohm’s law resistivity. In a systematic review of the samples from the spectra, initially, with increasing the applied voltage, the proposed composites slowly increased; then, when elevated to higher values, the current increased and resistance decreased. As seen in Figure 14b, the *lnI-lnV* spectra establishes the considered films’ nonlinearity and degree of responsibility for the conduction mechanism. Figure 14c,d illustrates the two different regions that exist, which are indicated as regions (*I*) and (*II*). Therefore, the claimed nonlinearity has two possible reasons: the first one is the ability of the applied voltage to get through the nominal voltage, which mainly decreases the sample’s resistance; thus, the conductance and the current increase [90]. Optically, a narrow region on the energy bandgap allows for more carrier charge between the levels and coordinates with an increased current from *nA* to *mA*. The other argument suggests developing the conduction route while adding the PB-Nd^+3^ content to the PVA/PVP polymeric matrix, which causes an increase in the current values [90]. The attained electrical results suggest that the proposed Nd^+3^-doped PVA/PVP polymeric composites are a promising candidate for varistors devices. Detailed examination of the considered polymeric composites was in-line with the previously published work by H. Elhosiny Ali et al. The electrical characterization of PVAL flexible composites was affected by Ru-metal dopants [90].

## 4. Conclusions

The low-cost solution casting technique offers the successful synthesis of PB-Nd^+3^-doped PVA/PVP composite films. Furthermore, the XRD study confirmed the semi-crystalline nature of the blend polymer. FT-IR analysis performed on the blend-polymer production confirmed the complexity of the PB-Nd^+3^ composite films. The optical absorption offered no evidence of the UV-region; in contrast, the transmission dropped along with the increased PB-Nd^+3^ content. The direct and indirect bandgap calculated using ASF and Tauc’s equation showed slightly reduced values, according to the high doping content of PB-Nd^+3^ composite films. The E_u_ values were estimated from the absorption coefficient, which ranged from 5.41 eV to 12.32 eV, where the high values cause the dysfunction of the composite preparation, which may contribute to the bandgap’s local state. The refractive index concerning the bandgap was evaluated using the seven suggested theoretical equations. The refractive index values were likely to increase with the addition of PB-Nd^+3^ doping on the PVA/PVP polymeric blend matrix. The nonlinear optical parameters reconstructed from the bandgap depict a noticeable increase and the influence of PB-Nd^+3^ concentrations. The optical limiting effects of the composite films were enhanced and attenuated, as PB-Nd^+3^ increased.

The real and imaginary parts of the dielectric respond to the inclusion of PB-Nd^+3^ content. Carrier mobility and imperfections may exist on the real dielectric permittivity. Meanwhile, the imaginary dielectric permittivity revealed increased ionic-carrier and conductivity behavior. In addition, the relation of dielectric, corresponding to the wt% of PB-Nd^+3^, suggested an instant impact on the energy storing of the Nd_2_O_3_:PVA/PVP composite films. The absence of a relaxation peak on the $M^{\prime \prime }$ of the electric modules at lower frequency regions indicates an interaction between PB-Nd^+3^ and the blend matrix. In addition, AC conductivity affirmed composite dispersion as they increase with a high concentration of PB-Nd^+3^ dopants, which indicates a higher number of free ions. The s exponent obeyed the hopping system, where the I-V nonlinear characteristics results indicate the films as possible candidates for varistors. Moreover, the PVA/PVP blend polymer doped with different wt% PB-Nd^+3^ films can be used in many potential applications, such as optoelectronics, cut-off lasers, and electrical devices.

## Figures and Tables

**Table 1:** Optical energy bandgap of the PVA/PVP polymer blend doped with the PB-Nd^+3^ composite films.

Samples	E_g (ind)_, (eV) (the Indirect Bandgap)	E_g (d)_, (eV) (the Direct Bandgap)	E_u,_ (eV) (Urbach’s Tail)
***PB-Nd^+3^-0***	5.6	6.09	5.42
***PB-Nd^+3^-1***	5.49	6.07	4.66
***PB-Nd^+3^-2***	5.52	6.05	6.474
***PB-Nd^+3^-3***	5.36	6.005	8.69
***PB-Nd^+3^-4***	5.36	5.95	7.88
***PB-Nd^+3^-5***	4.82	5.83	12.33

**Table 2:** The refractive index values obtained from Moss, Ravindra, Hervé, Reddy, Anani, Kumar- Singh, and Hosam–Ibrahim–Heba relations and the average refractive index parameters for the investigated PVA/PVA blend polymeric films with various Nd_2_O_3_ additions for (**a**) direct band transition and (**b**) indirect band transition.

Samples	(a) Refractive Index (*n*) Values Using Direct Band Transition							
	Moss Relation	Ravindra et al. Relation	Herve and Vandamme Relation	Reddy et al. Relation	Anani et al. Relation	Singh-Kumar Relation	Hosam—Ibrahim—Heba Relation	Mean Values
***PB-Nd^+3^-0***	1.987	2.052	1.747	1.785	2.182	1.880	1.714	1.907
***PB-Nd^+3^-1***	1.988	2.053	1.749	1.788	2.186	1.882	1.717	1.909
***PB-Nd^+3^-2***	1.990	2.055	1.752	1.791	2.19	1.884	1.719	1.912
***PB-Nd^+3^-3***	1.994	2.059	1.758	1.799	2.199	1.889	1.724	1.917
***PB-Nd^+3^-4***	1.998	2.0640	1.765	1.808	2.21	1.894	1.731	1.924
***PB-Nd^+3^-5***	2.009	2.074	1.780	1.828	2.234	1.907	1.745	1.939
**Samples**	**(b) Refractive Index *(n)* Values Using Indirect Band Transition**							
	**Moss Relation**	**Ravindra et al. Relation**	**Herve and Vandamme Relation**	**Reddy et al. Relation**	**Anani et al. Relation**	**Singh-Kumar Relation**	**Hosam** **—Ibrahim** **—Heba** **Relation**	**Mean Values**
***PB-Nd^+3^-0***	2.029	2.095	1.812	1.869	2.28	1.932	1.773	1.970
***PB-Nd^+3^-1***	2.039	2.106	1.827	1.888	2.302	1.944	1.787	1.985
***PB-Nd^+3^-2***	2.036	2.103	1.823	1.883	2.296	1.941	1.783	1.981
***PB-Nd^+3^-3***	2.051	2.118	1.846	1.912	2.328	1.959	1.804	2.003
***PB-Nd^+3^-4***	2.051	2.118	1.846	1.912	2.328	1.959	1.804	2.003
***PB-Nd^+3^-5***	2.107	2.175	1.933	2.019	2.436	2.027	1.880	2.082

**Table 3:** Nonlinear calculated optical values for direct and indirect bandgaps of the various systems. (**a**) Direct bandgap of the various systems. (**b**) Indirect bandgap of the various systems.

(a)					
Samples	High-Frequency Dielectric Constants, (*ɛ_∞_*)	Static Dielectric Constant, (*ɛ_o_*)	$\chi^{\left(1\right)}$,	$\chi^{\left(3\right)}$ **,** **10^−13^** **(esu)**	$n_{\left(2\right)}$ **,** **10^−11^** **(esu)**
***PB-Nd^+3^-0***	3.636	630.497	0.209	3.30	0.65
***PB-Nd^+3^-1***	3.646	621.577	0.210	3.35	0.66
***PB-Nd^+3^-2***	3.655	612.743	0.211	3.399	0.669
***PB-Nd^+3^-3***	3.677	593.172	0.213	3.51	0.689
***PB-Nd^+3^-4***	3.704	569.825	0.215	3.653	0.715
***PB-Nd^+3^-5***	3.76	521.035	0.220	3.984	0.773
**(b)**					
**Samples**	**High-Frequency Dielectric Constants, (*ɛ_∞_*)**	**Static Dielectric Constant,** **(*ɛ_o_*)**	$\chi^{\left(1\right)}$ **,**	$\chi^{\left(3\right)}$ **,** **10^−13^** **(esu)**	$n_{\left(2\right)}$ **,** **10^−11^** **(esu)**
***PB-Nd^+3^-0***	3.882	435.488	0.229	4.712	0.9
***PB-Nd^+3^-1***	3.940	398.133	0.234	5.110	0.97
***PB-Nd^+3^-2***	3.924	408.099	0.2328	4.998	0.95
***PB-Nd^+3^-3***	4.012	356.832	0.239	5.627	1.058
***PB-Nd^+3^-4***	4.012	356.832	0.239	5.627	1.058
***PB-Nd^+3^-5***	4.337	215.932	0.265	8.478	1.533

**Table 4:** Values of *DC* electrical conductivity *σ_DC_* determined from the lower frequency fit of *σ_AC_* spectra to the power law and their corresponding fractional exponent *s*.

Samples	σ_DC_, (S/m)	*s*
**PB-Nd^+3^-0**	6.750 × 10^−11^	1.00633
**PB-Nd^+3^-1**	1.184 × 10^−10^	1.00219
**PB-Nd^+3^-2**	1.060 × 10^−10^	1.00299
**PB-Nd^+3^-3**	1.520 × 10^−10^	1.00204
**PB-Nd^+3^-4**	2.088 × 10^−10^	0.99926
**PB-Nd^+3^-5**	4.220 × 10^−10^	0.99656

## Data Availability

The data presented in this study are available on request from the corresponding author.
